# Ethanol- and/or Taurine-Induced Oxidative Stress in Chick Embryos

**DOI:** 10.1155/2013/240537

**Published:** 2013-03-21

**Authors:** Emily J. Berning, Noah Bernhardson, Kelly Coleman, Dina A. Farhat, Courtney M. Gushrowski, Alison Lanctot, Benjamin H. Maddock, Kathryn G. Michels, Luke A. Mugge, Catherine M. Nass, Sarah M. Yearsley, Robert R. Miller

**Affiliations:** ^1^School of Dentistry, Indiana University, Indianapolis, IN 46202, USA; ^2^Biology Department, Hillsdale College, Dow 213, 278 N. West Street, Hillsdale, MI 49242-1205, USA; ^3^Neurology Department, Northwestern University, Chicago, IL 60611, USA; ^4^Chicago College of Osteopathic Medicine, Downers Grove, IL 60515, USA; ^5^U.S. Army Criminal Investigation Laboratory, Forest Park, GA 30297, USA

## Abstract

Because taurine alleviates ethanol- (EtOH-) induced lipid peroxidation and liver damage in rats, we asked whether exogenous taurine could alleviate EtOH-induced oxidative stress in chick embryos. Exogenous EtOH (1.5 mmol/Kg egg or 3 mmol/Kg egg), taurine (4 *μ*mol/Kg egg), or EtOH and taurine (1.5 mmol EtOH and 4 *μ*mol taurine/Kg egg or 3 mmol EtOH and 4 *μ*mol taurine/Kg egg) were injected into fertile chicken eggs during the first three days of embryonic development (E_0–2_). At 11 days of development (midembryogenesis), serum taurine levels and brain caspase-3 activities, homocysteine (HoCys) levels, reduced glutathione (GSH) levels, membrane fatty acid composition, and lipid hydroperoxide (LPO) levels were measured. Early embryonic EtOH exposure caused increased brain apoptosis rates (caspase-3 activities); increased brain HoCys levels; increased oxidative-stress, as measured by decreased brain GSH levels; decreased brain long-chain polyunsaturated levels; and increased brain LPO levels. Although taurine is reported to be an antioxidant, exogenous taurine was embryopathic and caused increased apoptosis rates (caspase-3 activities); increased brain HoCys levels; increased oxidative-stress (decreased brain GSH levels); decreased brain long-chain polyunsaturated levels; and increased brain LPO levels. Combined EtOH and taurine treatments also caused increased apoptosis rates and oxidative stress.

## 1. Introduction

Exogenous ethanol (EtOH) causes elevated brain and hepatic homocysteine (HoCys) levels, decreased brain and hepatic taurine levels, and increased apoptosis rates within embryonic chick brains and livers [1–3]. Exogenous EtOH and exogenous HoCys are both teratogenic in chick embryos. Exposure to either teratogen causes reduced brain masses, elevated brain lipid hydroperoxide (LPO) levels, elevated brain membrane lipid peroxidation intermediates, and elevated brain caspase-3 activities [4–8].

**Figure 1:**
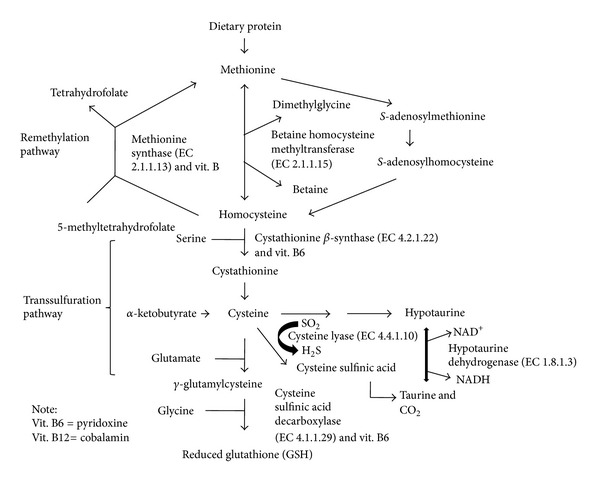
Homocysteine removal via the remethylation and transsulfuration pathways with reference to glutathione and taurine synthesis.

HoCys catabolism uses remethylation pathways and the transsulfuration pathway (Figure 1). In remethylation pathways, HoCys is remethylated back to methionine by using either betaine homocysteine methyltransferase (EC 2.1.1.15; non-folate-dependent remethylation), or the cobalamin-dependent enzyme, methionine synthase (EC 2.1.1.13), which uses 5-methyltetrahydrofolate as the methyl donor [9]. In the transsulfuration pathway, HoCys is converted to cystathionine through the use of cystathionine *β*-synthase (EC 4.2.1.22) and cystathionine is ultimately converted into *α*-ketobutyrate, reduced glutathione (GSH), or taurine [9, 10] (Figure 1).

EtOH-induced decreased taurine levels within embryonic chick brains [2, 3] may be devastating because taurine's oldest known function is regulating osmotic stress [11]. During mammalian brain swelling, astrocytes release taurine into extracellular spaces creating a hypertonic environment and cause H_2_O efflux from brain cells [11, 12]. Hypotonicity causes astrocytes and neurons to incorporate cysteine sulfinic acid and synthesize taurine through the use of cysteine sulfinic acid decarboxylase (CSAD; EC 4.1.1.29) [11, 13]. Ironically, elevated levels of *D/L*-homocysteine, *L*-cysteate, *L*-glutamate, and *L*-aspartate are all potent inhibitors of Na^+^-dependent cysteine sulfanilic acid uptake in chick astrocytes, chick neurons, and rat astrocytes [13]. Hence, EtOH-induced reductions in brain taurine levels coupled with EtOH-induced increased brain HoCys levels [1–3] may alter neural osmotic regulation [11, 13].

Taurine, and especially hypotaurine, has membrane-protective and antioxidant effects in a variety of systems [11, 14, 15]. Taurine supplementation ameliorated EtOH-induced increased lipid peroxidation, alcohol-induced liver damage, and EtOH-induced cytochrome p450-2E1 (CYP 2E1) activities within Sprague-Dawley rats [16]. Because cats have extremely low CSAD activities and primates have low brain CSAD activities, dietary taurine is essential for cats and is conditionally essential for primates [11, 14, 15, 17]. Taurine deficiencies in cats cause destabilization and fragmentation of retina and *tapetum* membranes; increased frequencies of retinopathy and blindness; increased frequencies of spontaneous abortions and stillbirths; altered immune systems where the proportions, morphology, and functions of leukocytes are altered; and myocardial failure (dilated cardiomyopathy) [11, 14, 15, 18].

Neuroblasts and glioblasts cell division and migration within the cortex and cerebellum of cat brains is normally completed by the third neonatal week [18]. In taurine-deprived kittens, cell division and migration within the cortex and cerebellum is delayed [15, 18]. Taurine supplementation ameliorated reduced synaptic connections, reduced neuron and glial cell numbers, and increased apoptosis rates within the cortex of fetal rat brains experiencing intrauterine growth restriction [19]. Consequently, taurine is important in embryonic and neonatal development.

Because EtOH reduced taurine levels within embryonic chick brains [2, 3], the overall objective of this study was to determine if taurine supplementation alleviated EtOH-induced oxidative stress within embryonic chick brains. Oxidative-stress measures included rates of apoptosis (caspase-3 activities), homocysteine (HoCys) levels, reduced glutathione levels (GSH), membrane fatty acid composition, and lipid hydroperoxide (LPOs) levels. Unlike mammalian embryos, avian embryos are closed systems (cleidoic egg) that offer the ability to solely observe embryonic responses to a teratogen as compared to combined maternal responses, fetal responses, and maternal to fetal transport as seen within eutherian embryos.

## 2. Materials and Methods

All experiments used fertile, specific pathogen-free white leghorn chicken eggs (*Gallus domesticus*) purchased from a commercial hatchery. Chickens raised at this commercial hatchery ingest a standardized diet causing minimal differences in yolk composition from generation to generation. Animal care was in compliance with the *Guide for the Care and Use of Laboratory Animals, 8th Edition *(National Academies Press, Washington DC, USA, 2011). 

Control eggs were injected with approximately 25 *μ*L of H_2_O or a 1 : 1 (v/v) mixture of 0.01 M taurine and H_2_O (4 *μ*mol taurine/Kg egg) into each air sac during the first three days of development (*E*_0–2_). Experimental eggs were injected with approximately 25 *μ*L of a 1 : 1 (v/v) mixture of 47.5% EtOH and H_2_O, (1.5 mmol EtOH/Kg egg); a 1 : 1 (v/v) mixture of 95% EtOH and H_2_O (3 mmol EtOH/Kg egg); a 1 : 1 (v/v) mixture of 47.5% EtOH and 0.01 M taurine (1.5 mmol EtOH/Kg egg and 4 *μ*mol taurine/kg egg); or a 1 : 1 (v/v) mixture of 95% EtOH and 0.01 M taurine (3 mmol EtOH & 4 *μ*mol taurine/Kg egg) into each air sac during the first three days of development (*E*_0–2_). All embryos were incubated in a forced air incubator at 37.5°C and turned every 4 hours with relative humidity ranging from 80 to 90%. At 11 days of incubation [20, theoretical stage 37], blood was drawn from chorio-allantoic blood vessels and serum collected by centrifugation (1,000 ×g). Chick embryos were then decapitated, brains excised, and all serum and isolated brains stored at −80°C, until subsequent biochemical analyses. Because chicks normally hatch at 21 days of development, embryos at 11 days of development [20, theoretical stage 37] have completed approximately 52% of total embryogenesis (midembryogenesis) and embryonic exposure to EtOH and/or taurine during the first three days of development (*E*_0–2_) represents approximately 14% of embryogenesis.

Theoretical stage 37 [20, 11 days of development] was selected because exogenous EtOH (3 mmol EtOH/Kg egg) caused elevated brain HoCys levels; increased brain caspase-3 activities; increased brain lipid hydroperoxide levels (LPOs); decreased brain membrane polyunsaturated fatty acids (PUFAs) levels; and decreased brain taurine levels at theoretical stage 37 [1–3, 6–8]. The 4 *μ*mol of exogenous taurine/Kg egg dosage was recently deemed to be “semi-safe.” This dosage failed to impact brain masses, brain caspase-3 activities, or brain lipid hydroperoxide (LPO) levels within chick embryos at 18 days of development [20, theoretical stage 44] (unpublished data).

### 2.1. Caspase-3 Activities

In order to monitor apoptosis within brain homogenates, caspase-3 activities were measured using caspase-3 cellular activity assay kits. Embryonic chick brains were homogenized in 400 *μ*L of ice-cold 50 mM HEPES [4-(2-hydroxyethyl)-piperazineethanesulfonic acid] buffer (pH 7.4), 1 mM DTT (dithiothreitol), 0.1 mM EDTA (disodium ethylenediaminetetraacetic acid), and 0.1% CHAPS (3-[(3-cholamidopropyl) dimethylammonio]-1-propanesulfonate), and nonsoluble material was removed by centrifugation at 10,000 ×g (10 min at 4°C). Aliquots from each supernatant were assayed for total protein [21] and caspase-3 activity [5, 22]. As the fluorogenic acetylated-aspartate-glutamate-valine-aspartate-7-amino-4-methylcoumarin (AcDEVD-AMC) substrate was cleaved by activated caspase-3, the liberated AMC (7-amino-4-methylcoumarin) was detected by fluorescence at an excitation wavelength of 360 nm and an emission wavelength of 460 nm. Caspase-3 activities are reported as units of caspase-3/mg protein, and one unit of caspase-3 is defined as the release of 1 pmol of AMC liberated per minute from the 0.3 mM AcDEVD-AMC substrate at 30°C.

### 2.2. Brain HoCys Levels

In order to study the effects of exogenous EtOH and/or taurine on brain HoCys levels, the isolation and quantification of HoCys was achieved by modifying the technique of Yi et al. [23]. Embryonic brains were homogenized in 500 *μ*L of ice-cold 50 mM HEPES buffer (pH 7.4), 1 mM DTT, and 0.1 mM EDTA, and nonsoluble material was removed by centrifugation at 10,000 ×g (5 min at 4°C). Supernatants were deproteinated by the addition of 400 *μ*L of 0.4 M perchloric acid (PCA). Protein-containing precipitates were removed by centrifugation at 10,000 ×g (5 min at 4°C) and the pH was readjusted to approximately 6.5 to 7.5 by adding 15 *μ*L of 6 M NaOH to each supernatant.

HoCys and methionine derivatives were prepared as previously described [1, 22, 23]. The separation and quantification of HoCys derivative was achieved by HPLC using a 15 cm × 4.6 mm Discovery Bio-Wide Pore C 18 HPLC column (5 *μ*m beads) using a high-pressure liquid chromatography system (HPLC) equipped with a fluorescence detector. The mobile phase was 50 mM sodium monophosphate, 1 mM octane sulfonic acid, and 2% acetonitrile and adjusted to pH 2.7 with 85% phosphoric acid. The isocratic flow rate was 1 mL/min (1800 to 2100 PSI) and the excitation wavelength was 230 nm and the emission wavelength was 418 nm. Using these HPLC conditions, the retention time of HoCys was 2.254 ± 0.037 min [22]. HoCys concentrations within biological samples were determined from a standard curve prepared from known concentrations of *D/L*-homocysteine (0 to 10 *μ*mol HoCys).

### 2.3. Glutathione (GSH) Levels

Effects of exogenous EtOH and/or taurine on reduced glutathione (GSH) levels within embryonic chick brains utilized the protocols of Anderson [24, 25]. Embryonic brains were homogenized in 500 *μ*L of ice-cold 50 mM sodium phosphate buffer (pH 7.4) and 0.1 mM EDTA, and nonsoluble material was removed by centrifugation at 10,000 ×g (5 min at 4°C). Aliquots from each supernatant were assayed for total protein [21]. An additional aliquot of 50 *μ*L was removed from each supernatant and deproteinated by adding 190 *μ*L of 4% 5-sulfosalicylic acid and the protein-containing precipitate removed by centrifugation at 15,000 ×g (5 min at 4°C). An aliquot of 200 *μ*L was then removed and 1 mL of Ellman's reagent (0.1 mM 5,5′-dithio-bis(2-nitrobenzoic acid) in 0.1 M sodium phosphate buffer, pH 8.0) was added to each aliquot and incubated at 37.5°C for 30 min. As reduced GSH was converted into oxidized GSSG (oxidized glutathione disulfide), 5,5′-dithiobis(2-nitrobenzoic acid) was converted into 2-nitro-5-thiobenzoic acid, and the absorbance was measured at 412 nm. Absorbance values were linear when GSH levels ranged from 0 to 5000 pmol.

### 2.4. Serum Taurine Levels

Serum taurine levels were measured by modifying the technique of Waterfield [26]. Serum aliquots of 50 *μ*L were deproteinized by adding 50 *μ*L of cold 0.2 M 5-sulfosalicylic acid to each sample, and nonsoluble material removed by centrifugation at 11,500 ×g (2 min at 4°C). Columns containing 1 mL of Dowex 1 × 4 resin over 3 mL of Dowex-50W-8 resin in 12 mL reservoirs fitted with 1.5 cm frits were prewashed with 12 mL of 1 M HCl. After 40 *μ*L of 100 *μ*M homoserine was added to each supernatant as an internal standard, supernatants were layered onto a dual-bed column and taurine and homoserine were eluted with 4 mL of H_2_O. OPA (*ortho*-phthalaldehyde) derivatives were synthesized according to Waterfield's approach [26] and filtered through 0.2 *μ*m Millex-FG13 PTFE filters.

Aliquots of 20 *μ*L were injected onto a 15 cm × 4.6 mm Discovery Bio-Wide Pore C 18 HPLC column (5 *μ*m beads) on a SCM 1000 high-pressure liquid chromatography system (HPLC) equipped with dual detectors including a fluorescence detector and a UV-VIS detector. The UV-VIS detector was set at a wavelength of 250 nm and the excitation wavelength of the fluorescence detector was 350 nm with an emission wavelength of 476 nm [2, 3]. The mobile phase was 0.05 M NaH_2_PO_4_ (pH 5.4) mixed with a 1 : 1 solution of methanol-water (43 : 57, v/v). The isocratic flow rate was 1 mL/min (1900 to 2100 PSI). Using this protocol, the retention time of homoserine (internal standard) was 3.683 ± 0.299 min and the retention time of taurine was 4.504 ± 0.287 min. 

### 2.5. Relative Brain Membrane Fatty Acid Levels

Because reactive oxygen species (oxygen radicals (O_2_^−∙^) and hydroxyl radicals (HO^−∙^)) can attack any unsaturated neutral lipid or any unsaturated phospholipid [27], it became imperative to determine if exogenous EtOH and/or taurine altered brain membrane fatty acid composition. Embryonic brains were homogenized in 400 *μ*L of ice-cold 50 mM HEPES buffer (pH 7.4), 1 mM DTT, and 0.1 mM EDTA, and total lipids were extracted by the Folch technique [28]. Samples were concentrated by the evaporation of solvent under N_2_ and stored overnight at −40°C.

Neutral lipids were separated from membrane lipids on silicic acid columns [4–6, 29]. Liquid chromatography columns (12 mL reservoirs) were packed with anhydrous 100–200 mesh SIL-R to a bed height of 3.5 cm. After columns were washed several times with chloroform, total lipids were dissolved in 0.5 mL of 2 : 1 chloroform-methanol (v/v) and applied to a column. Neutral lipids were eluted with 4 mL of chloroform, and membrane lipids, including phospholipids and some glycolipids, were eluted with 4 mL of methanol. After fractionated samples were dried under N_2_, membrane lipid samples were saponified and liberated fatty acids were methylated according to Metcalfe et al. [30].

Fatty acid methyl esters (FAMEs) were separated on an Omega-wax capillary gas chromatography (GC) column (30 m × 0.53 mm × 0.25 *μ*m film thickness) in a GC. Injector temperature was 225°C and the flame ionization detector was set at 250°C with a He carrier gas rate of 20–25 mL/min. The initial column temperature was 185°C. After a 5 min delay, the column temperature was increased to 205°C at a rate of 2°C/min. The split ratio was 3 : 1. After methyl pentadecanoate was added as an internal standard (48.75 nmol/sample), individual FAMEs within each biological sample were identified by their retention times, quantified as compared to the internal standard, and data expressed as %mol.

### 2.6. Brain Lipid Hydroperoxide Levels

Lipid hydroperoxides (LPOs) are peroxidation intermediates [27] and were extracted by homogenizing brains in 500 *μ*L of ice-cold 50 mM HEPES buffer (pH 7.4), 1 mM DTT, and 0.1 mM EDTA. Samples were deproteinated by adding 500 *μ*L of *meta*-phosphoric acid (20 mg/mL in methanol) to each sample, and protein-containing precipitates were removed by centrifugation (5,000 ×g for 5 min). Nonpolar lipid hydroperoxides were extracted from each supernatant by adding 3 mL of chloroform, followed by centrifugation for 5 min at 2,000 ×g. An aliquot of 1 mL was removed from each organic layer and spectrophotometrically assayed for LPOs by using lipid hydroperoxide assay kits. LPOs are highly unstable and readily promote the conversion of Fe^+2^ to Fe^+3^ and were detected by using thiocyanate ions as the chromogen [31].

### 2.7. Statistical Analyses

Membrane fatty acid composition is reported as %mol. Because percentages may form binomial distributions rather than normal distributions, all %mol data were subjected to arcsine transformations prior to analysis of variance (ANOVA) followed by post hoc Tukey tests (honestly significant difference tests). Other data sets were not subjected to arcsine transformation before ANOVA analyses were followed by post hoc Tukey tests (Honestly Significant Difference tests). The significance level in post hoc Tukey tests was *P* ≤ 0.05.

## 3. Results and Discussion

### 3.1. Embryo Viability (Brain Masses and Brain Caspase-3 Activities)

Early embryonic exposure to EtOH (3 mmol/Kg egg) and taurine (4 *μ*mol/Kg egg) caused approximately a 1.8-fold and a 1.6-fold decrease in brain masses at 11 days of development, respectively (Table 1). While the combination of exogenous EtOH and taurine caused reduced brain masses, the reductions were insignificant. Brain caspase-3 activities increased by approximately 1.8-fold when exposed to either 1.5 mmol EtOH/Kg egg or 3 mmol EtOH/Kg egg as compared to controls. Meanwhile, exposure to 4 *μ*mol of exogenous taurine/Kg egg caused approximately a 1.7-fold increase in brain caspase-3 activities as compared to controls. The combined treatments of EtOH and taurine (1.5 mmol EtOH and 4 *μ*mol taurine/Kg egg or 3 mmol EtOH and 4 *μ*mol taurine/Kg egg) also caused increased brain caspase-3 activities as compared to controls (Table 1). Hence, EtOH and/or taurine caused increased brain apoptosis rates.

Early embryonic EtOH (3 mmol/Kg egg) exposure (*E*_0–2_) caused increased brain and hepatic caspase-3 activities and reduced brain masses at 11 days of development (midembryogenesis) [1–3, 6–8, 32]. Hence, these observations were verified in this paper. However, the observations that exogenous taurine also caused reduced brain masses and promoted increased brain caspase-3 activities are novel and indicate that exogenous EtOH and/or taurine are embryopathic.

In a preliminary study in this laboratory, a 50 mg of exogenous taurine/Kg egg dosage (400 *μ*mol taurine/Kg egg) during early embryogenesis (*E*_0–2_) caused reduced percentage of living chick embryos at 11 days of development (theoretical stage 37) to 31.9 ± 10.2% as compared to 86.7 ± 3.1% within controls (*t* = 10.80; df = 6; *P* ≤ 0.0001) (unpublished data). In this study, living embryos were defined as possessing a beating heart, and there were 4 rounds of injections (*N* = 4) with 18 to 21 eggs within each injection group. In this study, a correlation coefficient (*r*) of 0.52 (*F* = (1, 22) 8.20; *P* = 0.009) was observed when serum taurine levels were correlated to brain caspase-3 activities across all groups. This last observation indicates that exogenous taurine is more toxic in chick embryos at 11 days of development as compared to chick embryos at 18 days of development. The exogenous dosage of 4 *μ*mol taurine/Kg egg during the first three days of development previously failed to impact brain caspase-3 activities within 18-day chick embryos (late embryogenesis) and was, therefore, thought to be “semi-safe” (unpublished data). In this study, an exogenous dosage of 4 *μ*mol taurine/Kg egg during the first three days of development caused increased brain caspase-3 activities within 11 day chick embryos (midembryogenesis) (Table 1). Taurine-induced toxicity within chick embryos was first reported by Van Gelder and Belanger [33] who reported that a single injection of 100 *μ*mol taurine/50 g egg (2 mmol taurine/Kg egg) caused increased incorporation of taurine into developing hearts and brains at 15 days of development and was associated with ataxia, reduced muscle strength, a loss of motor coordination, and reduced embryo viability. Consequently, we have still not found a safe exogenous taurine dosage for chick embryos at 11 days of development (Tables 1, 2, and 3).

### 3.2. Brain Homocysteine (HoCys) Levels

Exogenous EtOH, taurine, and the combination of EtOH and taurine all caused increased brain HoCys levels (Table 1). Exposure to 3 mmol EtOH/kg caused a 1.9-fold increase in brain HoCys levels and exposure to 4 *μ*mol exogenous taurine/Kg egg caused a 2.3-fold increase in brain HoCys levels as compared to controls. The combined treatments of EtOH and taurine (1.5 mmol EtOH and 4 *μ*mol taurine/Kg egg and 3 mmol EtOH and 4 *μ*mol taurine/Kg egg) both caused elevated brain HoCys levels (Table 1). When brain caspase-3 activities were correlated to brain HoCys levels across all groups, Pearson's product moment (*r*) was 0.35 (*F* = (1, 47) 6.48; *P* = 0.0004). Thus, EtOH- and taurine-induced increased brain caspase-3 activities correlated to EtOH- and taurine-induced increased brain HoCys levels (Table 1). EtOH-induced and taurine-induced increases in brain HoCys Levels can promote the synthesis of reactive oxygen species (ROS). Two HoCys molecules can undergo autooxidation and form a dimer (homocysteine: oxidized disulfide) by liberating two hydrogen ions and two electrons. In doing so, hydrogen peroxide and hydroxyl radicals can be generated and thus produce reactive oxygen species, that is, hydroxyl radicals (HO^−∙^) and oxygen radicals (O_2_^−∙^) [34].

### 3.3. Brain GSH Levels

Early embryonic exposure to EtOH (1.5 mmol EtOH/Kg egg and 3 mmol/Kg egg) and taurine (4 *μ*mol/Kg egg) caused approximately a 1.2-fold to 1.3-fold decrease in brain GSH levels at 11 days of development, respectively (Table 1). The combined treatments of EtOH and taurine (1.5 mmol EtOH and 4 *μ*mol taurine/Kg egg or 3 mmol EtOH and 4 *μ*mol taurine/Kg egg) both caused approximately a 1.2-fold to 1.0-fold decrease in brain GSH levels as compared to controls. Total brain thiol (GSH and GSSG) levels were largely unaffected (Table 1). When brain caspase-3 activities were correlated to brain GSH levels within all groups, Pearson's product moment (*r*) was −0.29 (*F* = (1, 48) 4.40; *P* = 0.004). Thus, as apoptosis rates increased (brain caspase-3 activities), brain GSH levels decreased.

Recently, we demonstrated that EtOH (3 mmol/Kg egg) exposure during the first three days of development (*E*_0–2_) caused decreased brain selenium-dependent glutathione peroxidase (GPx; EC. 1.11.1.9) activities and non-selenium-dependent GPx activities at 11 days of development (midembryogenesis) (unpublished data) (*P* < 0.05). Glutathione peroxidases (GPx), both selenium-dependent and non-selenium-dependent, are well-known first-line defense antioxidant enzymes because they catalyze the reduction and ultimate removal of lipid hydroperoxides (LPOs), and other peroxides, as follows [35, 36]: 
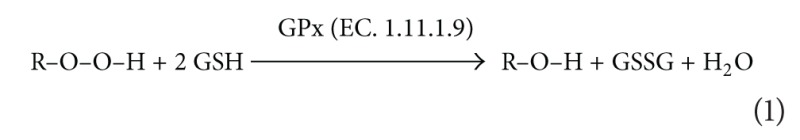
 Consequently, EtOH-induced reduction in brain GSH-dependent GPx activities could certainly contribute to oxidative stress and slow the catabolism of EtOH-induced increased LPO levels (Table 1).

### 3.4. Serum Taurine Levels

Increased endogenous serum taurine levels were caused by exogenous EtOH (1.5 mmol EtOH/Kg egg and 3 mmol/Kg egg), taurine (4 *μ*mol/Kg egg), and EtOH and taurine (1.5 mmol EtOH and 4 *μ*mol taurine/Kg egg and 3 mmol EtOH and 4 *μ*mol taurine/Kg egg) (Table 1). Early embryonic exposure to 1.5 mmol exogenous EtOH/Kg egg and 3 mmol exogenous EtOH/Kg egg caused approximately a 2.2-fold increase and a 3.1-fold increase in serum taurine levels, respectively. Meanwhile, exposure to 4 *μ*mol of exogenous taurine/Kg egg caused approximately a 2.4-fold increase in serum taurine levels. The combined treatments of EtOH and taurine (1.5 mmol EtOH and 4 *μ*mol taurine/Kg egg and 3 mmol EtOH and 4 *μ*mol taurine/Kg egg) both caused elevated serum taurine levels as compared to controls (Table 1). When serum taurine levels were correlated to brain caspase-3 activities within all groups, Pearson's product moment (*r*) was 0.61 (*F* = (1, 24) 11.54; *P* = 0.003) and when serum taurine levels were correlated to brain HoCys levels within all groups, Pearson's product moment (*r*) was 0.43 (*F* = (1, 24) 6.05; *P* = 0.003). Therefore, EtOH-induced and taurine-induced increases in serum taurine levels correlated to increased apoptosis rates and increased HoCys levels within embryonic chick brains.

Presumably, exogenous taurine caused increased endogenous serum taurine levels (Table 1) by diffusing into the yolk and then entering the embryo during vascularization. Previously, it was demonstrated that exogenous taurine exposure during the first three days of embryonic development (*E*_0–2_) caused increased endogenous chick serum taurine levels during late embryogenesis (18th day of development) (unpublished data). This last observation has now been extended to midembryogenesis (11 days of incubation; Table 1).

While it was not surprising that exogenous taurine caused increased serum taurine levels (Table 1), it was surprising to find that early exogenous EtOH exposure caused increased levels of serum taurine (Table 1). One hypothesis in explaining this phenomenon is EtOH-induced increases *de novo* taurine synthesis rates. Mammalian taurine synthesis uses cysteine dioxygenase (CDO: EC. 1.13.11.20) to catalyze the oxidation of cysteine to cysteinesulfonate. Cysteine sulfinic acid decarboxylase (CSAD: EC. 4.1.1.29) then catalyzes the decarboxylation of cysteine sulfinic acid to hypotaurine and taurine (Figure 1) [37]. Chick embryos can synthesize taurine from cysteine, and very high cysteine lyase (EC. 4.4.1.10) activities are found in the area vasculosa within 10-somite (approximately 33 hr) chick embryos [38]. Cysteine lyase catalyzes the synthesis of cysteine sulfinic acid through the following reaction: 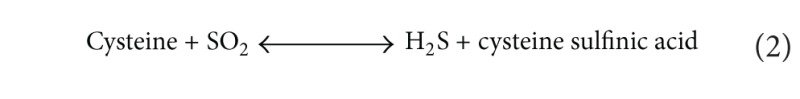


Chick neurons and glia readily incorporate cysteine sulfinic acid, and incorporated cysteine sulfinic acid is quickly converted into cysteic acid and is decarboxylated, via cysteine sulfinic acid decarboxylase (EC. 4.1.1.29), into taurine and hypotaurine [13].

Besides synthesizing taurine from cysteine, adult rats, adult chickens, and chick embryos also have the ability to synthesize taurine from serine [39–42]. In this very old evolutionary pathway, inorganic sulfate is added to ATP and converted to 3′-phosphoadenosine-5′-phosphosulfate (PAPS) followed by the transfer of the sulfate from PAPS to a hydroxylated organic compound, such as serine, to form taurine and is catalyzed by the pyridoxine-dependent enzyme, PAPS transferase (EC. 2.8.2) [43]. This pathway is illustrated as follows:

(3) ATP+SO4−2 →ATP-sulfurylaseadenosine-5′-phosphosulfate (APS)

(4) APS+ATP →APS  kinaseADP +3′-phosphoadenosine-5′-phosphate (PAPS)

(5) $$\begin{array}{c} \text{PAPS}+\text{serine}\overset{\text{PAPS}\;\text{transferase}}{\to }\text{taurine} \end{array}$$

We suspect that the hypothesis concerning exogenous EtOH-induced increased *de novo* taurine synthesis rates within chick embryos is false. Elevated *D/L*-HoCys levels are a potent inhibitor of Na^+^-dependent cysteine sulfanilic acid uptake into chick astrocytes and neurons [13] and should slow *de novo* brain taurine synthesis [11, 13]. EtOH-induced and taurine-induced increases in brain HoCys levels have been observed in this study (Table 1), and previous reports have demonstrated that exogenous EtOH (3 mmol/Kg egg) during the first three days of development (*E*_0–2_) caused reduced brain and hepatic taurine levels at 11 days of development [2, 3].

Thus, an alternative hypothesis predicts EtOH-induced increases in membrane fluidity and permeability may stimulate an efflux of taurine, and possibly K^+^, from neural cells into the circulatory system. EtOH-induced increases in membrane fluidity are documented [7, 27] and EtOH-induced and HoCys-induced decreased chick brain membrane phosphatidylcholine (PC) levels [5, 44], increased chick brain membrane phosphatidylethanolamine (PE) levels [5, 44], and decreased chick brain membrane PUFA levels [4, 6–8, 22, 27, 44] have been reported. EtOH-induced increases in taurine and PE levels within chick allantoic fluid have been reported and allantoic taurine and PE levels exceeded serum levels [45]. As cells lose cylindrically shaped phospholipids, such as PC, and gain triangularly shaped phospholipids, such as PE, membrane fluidity and permeability increase [7, 27, 46]. An EtOH- or HoCys-induced taurine efflux from brain and hepatic cells into the circulatory system could create hypertonic environments and promote cell shrinkage that is typically observed during apoptosis [11, 17]. In biopsies of adult human gliomas, histology revealed significant correlations between apoptotic cell density and taurine efflux in nonnecrotic (*r* = 0.73, *P* < 0.003) and necrotic (*r* = 0.63, *P* ⩽ 0.0005) biopsies and, thus, the authors argued that taurine efflux is a potential marker of apoptosis [47]. Increased extracellular taurine levels have been implicated as the mechanism in apoptosis-related cell shrinkage in several cell types [48–50] and p38 mitogen-activated protein kinase activation before taurine and K^+^ efflux have been associated with caspase-3 activation [50].

### 3.5. Relative Brain Membrane Fatty Acid Levels

Early embryonic EtOH exposure altered brain membrane fatty acid composition during midembryogenesis (Table 2). EtOH (3 mmol/Kg egg) caused a 2.2-fold reduction in brain membrane docosahexaenoic acid (22 : 6, n-3) levels; a 1.7-fold reduction in docosapentaenoic acid (22 : 5, n-6) levels; a 1.5-fold reduction in arachidonic acid (20 : 4, n-6) levels; a 2.2-fold reduction in linolenic acid (18 : 3, n-6) levels; and a 2.3-fold reduction in palmitolenic acid (16 : 3, n-3) levels as compared to controls (Table 2). Meanwhile, EtOH-induced increases in short-chain-saturated fatty acids were observed. EtOH caused a 1.5-fold increase in brain membrane palmitic acid (C16 : 0) levels and a 3.5-fold increase in myristic acid (14 : 0) levels when compared to controls (Table 2).

Early embryonic taurine (4 *μ*mol/Kg egg) exposure also altered brain membrane fatty acid composition during midembryogenesis (Table 2). Taurine caused a 1.4-fold reduction in brain membrane docosahexaenoic acid (22 : 6, n-3) levels and a 1.7-fold reduction in adrenic acid (22 : 4, n-6) levels as compared to controls. Meanwhile, taurine-induced increases in brain membrane linoleic acid (18 : 2, n-6) levels were observed. The combination of EtOH and taurine (3 mmol EtOH and 4 *μ*mol taurine/Kg egg) caused a 3.0-fold reduction in brain membrane docosahexaenoic acid (22 : 6, n-3) levels; a 1.6-fold reduction in docosapentaenoic acid (22 : 5, n-6) levels; a 2.2-fold reduction in adrenic acid (22 : 4, n-6) levels; a 1.4-fold reduction in arachidonic acid (20 : 4, n-6) levels; and a 1.2-fold decrease in palmitoleic acid (16 : 1, n-7) levels as compared to controls. Meanwhile, a 5.6-fold increase in brain membrane linoleic acid (18 : 2, n-6) levels was observed in EtOH- and- taurine-treated embryos as compared to controls (Table 2).

In order to look at EtOH-induced and taurine-induced changes in brain membrane fatty acid composition in a simplified and holistic manner, membrane fatty acid levels (Table 2) were converted into two ratios (Table 3). The first ratio was the sum of unsaturated/saturated membrane fatty acids (Σ unsaturated/Σ saturated fatty acids) and the second ratio was the sum of long-chain polyunsaturated fatty acids/short-chain membrane fatty acids (Σ long-chain PUFAs/Σ short-chain membrane fatty acids) (Table 3). In this study, long-chain PUFAs are defined as having hydrocarbon lengths ≥20 carbons, and short-chain fatty acids had hydrocarbon chain length ≤20 carbons. When Σ unsaturated/Σ saturated fatty acids ratios were correlated to Σ long-chain PUFAs/Σ short-chain membrane fatty acids in all groups, a Pearson's product moment (*r*) value of 0.37 (*F* = (1, 31) 4.99; *P* = 0.03) was observed. This correlation coefficient indicates that long-chain PUFAs are relatively more unsaturated as compared to short-chain fatty acids.

EtOH-treated embryos had a 1.5-fold decrease in Σ unsaturated/Σ saturated membrane fatty acid levels and a 1.9-fold decrease in Σ long-chain PUFAs/Σ short-chain membrane fatty acid levels as compared to controls (Table 3). Taurine-treated embryos and EtOH- and- taurine-treated embryos demonstrated insignificant differences in Σ unsaturated/Σ saturated membrane fatty acid levels as compared to controls. However, taurine-treated embryos had a 1.3-fold decrease in Σ long-chain PUFAs/Σ short-chain membrane fatty acid levels and EtOH- and- taurine-treated embryos had a 2.0-fold decrease in Σ long-chain PUFAs/Σ short-chain membrane fatty acid levels as compared to controls. Thus, EtOH-treated, taurine-treated-, and EtOH- and- taurine-treated embryos all demonstrated decreased levels of membrane long-chain PUFAs as compared to controls (Table 3).

### 3.6. Brain Lipid Hydroperoxide (LPOs) Levels

Because reduced PUFA levels are observed during lipid peroxidation [7, 10, 27], brain lipid hydroperoxides (LPOs) levels were measured (Table 3). LPOs are lipid peroxidation intermediates [27] and EtOH-treated embryos had a 5.0-fold increase in LPO levels, taurine-treated embryos had a 3.6-fold increase in LPO levels, and EtOH- and- taurine-treated embryos had a 4.8-fold increase in LPO levels as compared to controls (*P* < 0.05). When Σ long-chain PUFAs/Σ short-chain membrane fatty acid values were correlated to brain LPO levels within all groups, a Pearson's product moment (*r*) of −0.78 (*F* = (1, 28) 42.86; *P* < 0.0001) was observed.

Taurine, and especially hypotaurine, has membrane-protective and antioxidant properties in a variety of systems undergoing oxidative-stress [11, 14, 15]. Taurine supplementation ameliorated EtOH-ameliorated lipid peroxidation, alcohol-induced liver damage, and EtOH-induced increased cytochrome p450-2E1 (CYP 2E1) activities within rats [16]. Consequently, EtOH-induced and/or HoCys-induced taurine efflux from the brain into the circulatory system [2, 3] may remove a powerful antioxidant from the neurons and/or astrocytes that are undergoing EtOH-induced oxidative stress (Tables 2 and 3).

EtOH-induced formation of reactive oxygen species via the oxidation of Fe^2+^, increased xanthine oxidase activities, and increased cytochrome p450-2E1 (CYP 2E1; EC 1.14.13n7) activities have been discussed [7, 27]. EtOH- and taurine-induced increased reactive oxygen species levels can attack long-chain PUFAs and momentarily create LPO intermediates which cleave long-chain PUFAs into less saturated membrane fatty acids and produce a number of cytotoxic reactive aldehydes (Tables 2 and 3) [27, 31]. While the sources of reactive oxygen species during membrane lipid peroxidation in taurine-treated chick embryos are unclear (Tables 2 and 3), the commonality between EtOH-induced membrane lipid peroxidation and taurine-induced lipid peroxidation (Tables 2 and 3) elevates brain HoCys levels as seen in both exogenous treatments (Table 1). Elevated HoCys levels may produce elevated ROS levels [34].

## 4. Conclusions

This paper has failed to demonstrate that an exogenous dosage of 4 *μ*mol taurine/Kg egg can ameliorate EtOH-induced lipid peroxidation, oxidative-stress, and apoptosis within embryonic chick brains during midembryogenesis. Both exogenous EtOH and exogenous taurine treatments during early embryogenesis (*E*_0–2_) caused increased apoptosis rates (increased caspase-3 activities), increased oxidative-stress (decreased GSH levels), and increased membrane lipid peroxidation (reduced long-chain PUFAs and increased LPO levels) within embryonic chick brains (Tables 1, 2, 3). EtOH-induced and taurine-induced increases in brain HoCys levels were associated with decreased brain GSH levels and brain taurine levels (Tables 1 and 2) and may indicate EtOH-induced and taurine-induced decreased metabolism via the transsulfuration pathway (Figure 1) that may contribute to elevated HoCys levels. Because increased brain HoCys levels can be a source of reactive oxygen species [34], elevated HoCys levels appear to be associated with increased rates of apoptosis, oxidative-stress, and lipid peroxidation within embryonic chick brains and possibly cause a taurine efflux from brain and hepatic cells into the blood and may contribute to apoptosis-related osmotic stress and cell shrinkage (Table 1) [2, 3].

While the exogenous dosage of 4 *μ*mol/Kg egg failed to impact brain masses, brain caspase-3 activities, or brain LPO levels within chick embryos at 18 days of development (late embryogenesis), this same dosage clearly promotes increased rates of membrane lipid peroxidation and apoptosis within embryonic chick brains at 11 days of development (midembryogenesis) (Tables 1, 2, 3). Increased exogenous taurine-induced toxicity, as seen in embryonic chick brains at midembryogenesis as compared to late-embryogenesis, may not be surprising because chick brain and hepatic glutathione-dependent glutathione peroxidases (GPx; EC. 1.11.1.9) activities increase by 1.4-fold to 2.8-fold when comparing embryos at midembryogenesis (11 days of development) to late-embryogenesis (18 days of development) [51]. That is, the protective capabilities of antioxidant enzymes are greater during later stages of development as compared to midembryogenesis [51].

While a lower exogenous taurine dosage may have antioxidant capabilities within chick brains during midembryogenesis as compared to the prooxidant capabilities of the 4 *μ*mol/Kg egg dosage used in this study, this paper calls for caution and more study. The consumption of alcohol mixed with “energy drinks” has become popular and a concern within European and North American communities [52, 53]. Taurine levels in “energy drinks” can range from 10 mg (79.9 *μ*mol) to 1000 mg (7990 *μ*mol). Thus, a pregnant woman, weighing from 140 pounds (63.5 Kg) to 180 pounds (81.6 Kg), who consumes a single drink of alcohol mixed with an energy drink that contains 1000 mg taurine (7990 *μ*mol), consumes EtOH mixed with an exogenous taurine dosage ranging from 97.9 *μ*mol/Kg to 125.8 *μ*mol/Kg. These dosages are from 25.5-fold to 31.5-fold higher than the 4 *μ*mol exogenous taurine/Kg egg dosage used in this study which caused apoptosis and membrane lipid peroxidation within chick brains at midembryogenesis. This is a concern and warrants caution because a truly safe exogenous taurine dosage within a human fetus is currently unknown.

## Figures and Tables

**Table 1:** Effects of exogenous ethanol (EtOH) and/or taurine (Tau) on brain masses, brain caspase-3 (casp-3) activities, brain homocysteine (HoCys) levels, brain glutathione (GSH) levels, total brain thiol levels, and endogenous serum taurine levels in chick embryos at 11 days of development.

Exogenous taurine injected at *E*_0−2_	mg brain	Units of brain Casp-3 per mg brain protein	*μ*mol brain HoCys per g brain	pmol GSH per *μ*g brain protein	pmol total thiols per *μ*g brain protein	Endogenous serum taurine levels (*μ*M)
Controls (dH_2_O)	395.9 ± 144.0	22.32 ± 9.15	361.20 ± 71.186	105.61 ± 19.01	125.06 ± 24.16	16.53 ± 6.17
	*N* = 14	*N* = 9	*N* = 10	*N* = 12	*N* = 12	*N* = 5
1.5 mmol EtOH/Kg egg	344.4 ± 101.9	41.00* ± 15.62	559.11 ± 93.53	87.40* ± 7.64	111.74 ± 63.60	36.76* ± 7.58
	*N* = 14	*N* = 13	*N* = 9	*N* = 12	*N* = 12	*N* = 4
3.0 mmol EtOH/Kg egg	221.3* ± 76.6	40.64* ± 9.71	697.00* ± 112.24	84.68* ± 12.20	120.59 ± 48.44	50.58* ± 10.55
	*N* = 14	*N* = 14	*N* = 8	*N* = 15	*N* = 15	*N* = 4
4 *μ*mol Tau/Kg egg (0.5 mg/Kg egg)	249.1* ± 94.4	37.29* ± 12.75	828.44* ± 120.19	79.40* ± 13.89	101.61 ± 22.03	39.49* ± 8.49
	*N* = 14	*N* = 13	*N* = 9	*N* = 12	*N* = 15	*N* = 8
1.5 mmol EtOH/Kg egg and 4 *μ*mol Tau/Kg egg	291.2 ± 136.2	38.25* ± 14.28	1,122.13* ± 206.98	87.96* ± 18.92	62.48* ± 39.36	33.59* ± 8.52
	*N* = 14	*N* = 16	*N* = 8	*N* = 12	*N* = 13	*N* = 4
3.0 mmol EtOH/Kg egg and 4 *μ*mol Tau/Kg egg	349.4 ± 115.1	41.77* ± 14.34	901.43* ± 426.13	85.10* ± 7.79	156.39 ± 62.35	46.37* ± 1.59
	*N* = 14	*N* = 10	*N* = 7	*N* = 8	*N* = 8	*N* = 4
ANOVA						
*F* =	3.93	3.03	16.61	4.69	5.27	10.86
df =	5, 79	5, 70	5, 46	5, 66	5, 70	3, 24
*P*≤	0.003	0.01	0.0001	0.001	0.0004	0.0001

Data presented as mean ± standard deviation.

Units of Casp-3: one unit of caspase-3 is defined as the release of 1 pmol of AMC liberated per minute from the 0.3 mM AcDEVD-AMC substrate at 30°C.

*Experimental group differs from controls at *P* ≤ 0.05.

**Table 2:** Effects of exogenous ethanol (EtOH) and/or taurine (Tau) on brain membrane fatty acid composition at 11 days of development (methanol fraction : phospholipids and some glycolipids). Fatty acids in % mol (mean ± standard deviation).

	14:0	16:0	16:1, n-7	16-3, n-3	18:0	18:1, n-9	18:2, n-6	18:3, n-6	18:3, n-3	20:4, n-6	20:4, n-3	22:4, n-6	22:5, n-6	22:6, n-3
H_2_O	0.59 ± 0.13	15.98 ± 1.80	1.72 ± 0.31	1.19 ± 0.37	12.6 ± 3.75	23.70 ± 3.53	2.21 ± 1.06	3.56 ± 1.48	0.65 ± 0.36	13.85 ± 1.58	1.02 ± 0.95	4.29 ± 1.66	3.69 ± 1.11	15.53 ± 2.42
	*N* = 11	*N* = 11	*N* = 11	*N* = 11	*N* = 11	*N* = 11	*N* = 11	*N* = 11	*N* = 11	*N* = 11	*N* = 11	*N* = 11	*N* = 11	*N* = 11
3 mmol EtOH/Kg egg	2.08* ± 2.38	24.0* ± 3.34	1.98 ± 0.70	0.52* ± 0.16	13.08 ± 3.44	24.74 ± 5.58	4.84* ± 1.64	1.61* ± 0.47	1.30 ± 0.71	9.58* ± 2.00	1.87 ± 1.49	4.62 ± 2.87	2.18* ± 0.97	7.03* ± 1.52
	*N* = 8	*N* = 8	*N* = 8	*N* = 8	*N* = 8	*N* = 8	*N* = 8	*N* = 8	*N* = 8	*N* = 8	*N* = 8	*N* = 8	*N* = 8	*N* = 8
4 *μ*mol Tau/Kg egg	0.59 ± 0.30	16.20 ± 1.35	2.34 ± 0.53	0.72 ± 0.40	14.78 ± 4.14	24.80 ± 2.87	6.28* ± 3.01	2.42 ± 0.86	2.42 ± 0.85	12.30 ± 1.94	2.27 ± 2.39	2.55* ± 0.76	3.22 ± 1.00	10.8* ± 2.67
	*N* = 6	*N* = 6	*N* = 6	*N* = 6	*N* = 6	*N* = 6	*N* = 6	*N* = 6	*N* = 6	*N* = 6	*N* = 6	*N* = 6	*N* = 6	*N* = 6
3 mmol EtOH and 4 *μ*mol Tau/Kg egg	0.46 ± 0.19	16.60 ± 3.22	1.48* ± 0.31	0.61 ± 0.46	14.92 ± 3.12	23.29 ± 2.33	12.39* ± 2.94	5.69 ± 4.92	1.64 ± 1.37	9.58* ± 3.36	3.80* ± 2.32	1.93* ± 1.26	2.32* ± 1.25	5.16* ± 2.48
	*N* = 8	*N* = 8	*N* = 8	*N* = 8	*N* = 8	*N* = 8	*N* = 8	*N* = 8	*N* = 8	*N* = 8	*N* = 8	*N* = 8	*N* = 8	*N* = 8
*F* =	4.04	16.85	3.83	4.02	0.93	0.29	42.27	5.56	1.72	7.26	4.55	4.72	9.03	35.06
df =	3, 29	3, 29	3, 29	3, 29	3, 29	3, 29	3, 29	3, 29	3, 29	3, 29	3, 29	3, 29	3, 29	3, 29
*P*≤	0.02	0.0001	0.02	0.02	0.44	0.29	0.0001	0.004	0.19	0.001	0.01	0.008	.0002	.0001

Note: *experimental group significantly differs from controls at *P*≤ 0.05.

**Table 3:** Effects of exogenous ethanol (EtOH) and/or taurine (Tau) on indices of membrane lipid peroxidation in embryonic chick brains at 11 days of development.

Treatments at *E*_0−2_	Σ unsaturated/Σ saturated membrane fatty acids^1^	Σ long-chain PUFAs/Σ short-chain membrane fatty acids^1, 2^	nmoles brain LPO per g brain
Controls (dH_2_O)	2.35 ± 0.31	0.63 ± 0.10	7.6 ± 1.8
	*N* = 11	*N* = 11	*N* = 9
3 mmol EtOH/Kg egg	1.56* ± 0.22	0.34* ± 0.08	38.1* ± 6.3
	*N* = 8	*N* = 8	*N* = 20
4 *μ*mol Tau/Kg egg	2.30 ± 0.63	0.47* ± 0.09	27.1* ± 5.0
	*N* = 6	*N* = 6	*N* = 5
3 mmol EtOH and 4 *μ*mol Tau/Kg egg	2.20 ± 0.59	0.31* ± 0.14	35.7* ± 8.0
	*N* = 8	*N* = 8	*N* = 19
ANOVA			
*F* =	5.78	17.91	52.05
df =	3, 29	3, 29	3, 49
*P*≤	0.003	0.0001	0.0001

Data presented as mean ± standard deviation.

*Experimental group significantly differs from controls at *P*≤ 0.05.

Note: ^1^membrane fatty acids within methanol fractions (phospholipids and some glycolipids). ^2^Long-chain membrane fatty acids are defined as possessing ≥ 20 carbons and short-chain fatty acids < 20 carbons.
